# Strangulated Hernia

**Published:** 1872-07

**Authors:** 


*Strangulated Hernia.*

In a lecture on the above-named subject, delivered at St. Bartholomew's Hospital, and published in the *British Medical Journal*, Sir James Paget remarked that in hospital and private practice together he had operated an hundred times for strangulated hernia, but that to obtain conclusions of real value it would need a tabulation of at least a thousand cases.

Generally speaking, in a case of hernia with signs of strangulation present, where reduction by ordinary means cannot be accomplished, an operation should at once be performed, in some cases, although the hernia is irreducible, the symptoms of strangulation are slight, obscure, or incomplete. It is an easy rule for all these cases that you should operate when strangulation is suspected; this rule you must avoid, and learn the hard one to discriminate the cases that require operation.

The irreducibility of the hernia is a fallacious sign of strangulation, and the presence of the other local signs even in a marked degree, is not decisive of strangulation, and is not sufficient to prove the need of operating when the remoter signs are not present. The local characters usually present in a strangulated hernia, and sometimes the remoter signs, may be imitated in an inflamed hernia, which is not strangulated. Generally, in the inflamed hernia, without strangulation, the local signs precede and greatly predominate over the remoter and general signs; while, in a hernia which is inflamed after becoming strangulated, the remoter and general signs will still predominate over the local, and the history will tell that they preceded. If these means of discrimination fail, you must operate if you cannot easily reduce the hernia; the risk of operating is small in comparison with that of waiting, for an inflamed and irreducible hernia may at any time become strangulated.

A hernia that has come down quickly, and the more it exceeds its usual size, the less is the probability of its being reduced without operation.

Again, the harder, more tense and painful a hernia is, the less the chance of reduction without an operation.

Again, if the remote and general signs of hernia are present and the hernia cannot be reduced, you must operate, or, if there be a swelling which may be a hernia, though it seem not likely to be a strangulated hernia, the operation must be performed at the seat of swelling.

If a patient have two herniæ that are irreducible and signs of strangulation, and you cannot tell which is strangulated, you must operate on both.

One or more actions of the bowels after symptoms of strangulation have set in, are of no weight against the propriety of operating; even frequent and regular action is not an absolute prohibition, as strangulation may involve only the omentum or only a part of the circumference of a portion of the intestine.

As a rule, while the bowels act you should not operate unless all the other signs of strangulation are well marked.

The sign we should most rely on as commanding the operation is vomiting. The rule is safe that recent irreducibility and vomiting are enough to justify the operation, even though there be no other signs of strangulation present. While there are notable kinds of vomiting characteristic of strangulated hernia, we should not be misguided by waiting for any particular kind. Any kind of vomiting, if it be repeated, is enough to justify operation in a hernia recently become irreducible.

Cessation of vomiting in the extreme condition of strangulated hernia is a token of evil rather than of good, if general improvement do not coincide with it. The pulse is 80 or 90 in a majority of ordinary cases in the early stages, and becomes more rapid as the symptoms of strangulation become more marked; the respirations usually are in due proportion to the pulse.

For the reduction of strangulated hernia without operation, Sir James Paget laid down the following general rules:

In cases, for instance, when the patient vomits fæcal matter and has peritonitis, or is in collapse, with a small rapid pulse, hiccough, or other such extreme signs, there should be no attempt at reduction without operation.

When the coverings of the hernia are so inflamed as to make it probable that sloughing or suppuration has taken place beneath them, reduction should not be attempted without operation; and even when less inflamed, none but slight and brief efforts at reduction should be made.

The longer the signs of strangulation have existed the shorter should be the efforts at reduction, but the intensity of pain in recent or acute hernia should not deter one from making the attempt.

In a hernia which has been habitually irreducible and becomes strangulated, you should operate at once. It is a safe rule of practice that, after a warm bath and a few hours rest in bed, a single attempt at reduction should be made; should this fail, chloroform or ether should be given, and then in some cases, but not in all, a second attempt made; this failing, the operation should be performed while the patient is still insensible.

The hot bath is useful in all cases that are not bad, unless in old and feeble persons; the patient should be simply soothed or relaxed in the bath, then wrapped in warm blankets, put into bed lying on his side or his back, with his knees drawn up, or with his pelvis a little raised, and then after an hour or two of complete rest the reduction attempted. The employment of rest and the bath is helped by opium when the hernia is painful. In the old, and others who may have had inactive bowels long before the strangulation, an enema of a large quantity of liquid should be used. Purgatives should not be used if there are marked symptoms of strangulation.

After the warm bath and rest have been tried, you may give chloroform or some other anæsthetic. In making the attempt at reduction you must be gentle and self-restraining, mindful of the delicacy of some of the structures you are handling, and that you may do them much more harm than would come of the operation which you are trying to arrest. These cautions are the more necessary because when the patient is under chloroform, you have nothing but your own sense and senses to tell you how far you may go without doing harm.

Chloroform is most useful in the herniæ of which the difficulty of reduction is chiefly due to muscular resistance, in the recent, or in the recently much enlarged; in the inguinal more than in the femoral; and in these more than in the umbilical; in the painful more than in the painless. In herniæ that have only recently come down, and are intensely painful, it is right to use chloroform or ether without waiting for the influence of the warm bath, but more commonly, if there be danger in waiting three or four hours, it is because strangulation is so far advanced that the operation ought to be performed without any previous attempts at reduction.

After the warm bath, rest and chloroform have been tried, and the reduction is not accomplished and strangulation exists, you should operate while the patient is still under the influence of chloroform; but if strangulation is not present you may wait, but must watch impatiently, for the hernia is likely soon to become strangulated. While waiting, ice or warm dressings, enemata, aperients or opiates may be used. Tobacco and curious postures, and shaking the legs up and the head down, and the cupping glasses, are more dangerous than the operation which they are intended to avert.

For doubtful or partial reduction there is one practical rule—operate if the symptoms of strangulation are not relieved. In cases in which reduction seems complete but the symptoms of strangulation are still present, operate, if you can feel a lump at or near the hernial ring.

Old age and disease may add to the risk of an operation for strangulated hernia, but they must be accepted. A patient must not be allowed to die with a strangulated hernia, if by any means whatever the strangulation can be relieved, and you must not be averted from the operation by the number of deaths that follow it.

The deaths after the operation may be 50 per cent., but the deaths due to the operation are not more than 2 or 3 per cent.

